# Serum alpha-1-antitrypsin level in the severity prognosis of systemic lupus erythematosus patients: Systematic exploration of novel biomarker

**DOI:** 10.37796/2211-8039.1297

**Published:** 2022-06-01

**Authors:** Supaporn Khamchun, Chunyanuch Thakaeng, Rattikan Na Lampang

**Affiliations:** a Department of Medical Technology, School of Allied Health Sciences, University of Phayao, Phayao 56000, Thailand; b Unit of Excellence in Integrative Molecular Biomedicine, School of Allied Health Sciences, University of Phayao, Thailand

**Keywords:** Biomarker, Protein, Serum, Prognosis, Systemic lupus erythematosus

## Abstract

**Background:**

Systemic lupus erythematosus (SLE) is a chronic autoimmune disease that affecting multi-organs injury and failure. The rapid, precision, and specificity prognosis by laboratory investigations could prevent active stage and severity in the disease.

**Aims:**

To systematically explore and investigate the candidate serum protein for development into the novel biomarker for severity prognosis of SLE patients.

**Methods:**

The proteins previously reported in abnormal level in serum/plasma of SLE patients since 2014–2020 were comprehensive collected. Thereafter, these serum proteins that found in other diseases were excluded. The association with molecules related to SLE severity of these candidate proteins were then predicted using bioinformatics STIRNG tool. The level of altered protein, which had the strong interaction to the severity molecules in serumof SLE patients was validated by Western blot, analyzed the correlation with anti-nuclear antigen (ANA) and performance of diagnosis, respectively.

**Results:**

From 26 collected serum/plasma proteins, alpha-1-antitrypsin protein was found the abnormal level in only SLE patients and strongly associated with severity molecules including C-reactive protein (CRP), complement C3, and C4. Additionally, the validation of serum alpha-1-antitrypsin in SLE patients exhibited the higher level than healthy controls and also had the positive correlation with ANA titer (r = 0.710). Furthermore, the area under ROC curve for diagnostic power of alpha-1-antitrypsin was 0.970 with 100% sensitivity and 90% specificity at cut-off 0.131/total serum protein.

**Conclusions:**

The higher level of alpha-1-antitrypsin in serum samples of SLE patients indicated as the novel biomarker for reliable and specific prognosis of disease severity.

## 1. Introduction

Systemic lupus erythematosus (SLE) is a highly-prevalent systemic autoimmune disease with chronic inflammation that results in severe and life-threatening illnesses. It can affect the global general population of all ages and both sexes, especially middle-aged (15–45 years old) women, who make up approximately 90% of all patients [1,2]. SLE is characterized by the production of autoantibodies which bind with nuclear (self) antigens via the pathogenic mechanism of self-tolerance abnormality and the activation of autoreactive lymphocytes. The binding of autoantibodies to nuclear antigens can cause the deposition of this immune complex in various organs and tissues such as skin, brain, kidney, lung, etc., leading to multi-organ damage and failure [3–5]. Currently, the diagnosis and prognosis of severity in SLE patients requires several laboratory investigations including the level of autoantibodies (anti-dsDNA, anti-Sm, etc.), complement C3, C4 in serum samples, and tissue biopsy for determination of immune complex accumulation in organs [6,7]. Nevertheless, the accuracy of this molecule detection requires a higher level in samples; patients have a severe or advanced stage of disease, and these investigations could not differentiate SLE from other diseases [8]. For reduction of this drawback, the new molecules in the serum/plasma of the patients, especially proteins, shall be applied as an alternative and/or supportive laboratory investigation for the reliability of diagnosis/monitoring of disease severity in organ tissues. Previously, studies of proteomic analysis had reported the abnormality of various serum/plasma protein levels in SLE patients, particularly the active stage, such as increased levels of haptoglobin [9], fibrinogen gamma chain [10], alpha-1-antitrypsin [11,12], apolipoprotein B [11,12], or the diminished level of apolipoprotein A-1 [9], histidinerich glycoprotein [10], clusterin [11], and albumin [12]. It is possible that several previous studies aimed to explore the altered serum/plasma proteins for serving as an alternative biomarker for the effective diagnosis and/or severity monitoring of SLE [13]. Nevertheless, a large number of the previously reported proteins remained not to be estimated the relationship with pathogenic mechanisms of SLE disease and exactly validated the level in the patient’s sample. In the present study, we gathered a comprehensive collection of the proteomic data that were previously published in medical and scientific databases. Subsequently, the collected serum/plasma proteins were chosen for further diagnosis/monitoring of SLE severity by the defined criteria specific to the patients and associated with various processes of disease severity using a bioinformatics tool. The serum level of the altered protein for a novel biomarker in SLE patients was compared to healthy controls by Western blot analysis, which determined the correlation with a titer of anti-nuclear antibody (ANA) testing, diagnostic performance with sensitivity and specificity values by statistical investigation. Accordingly, the results of this study could establish the ability of the altered serumprotein in SLE patients by systemic exploration and identification based an approach for development into a novel biomarker that had specificity and relation with the change of disease severity in multi-organ injury.

## 2. Methods

### 2.1. Subject selection and serum collection

The subjects in this study included ten participants who were SLE patients (age range: 20–45 years) according to sample size calculation (prevalence of the disease). These patients were diagnosed according to the European League Against Rheumatism (EULAR)/American College of Rheumatology (ACR) 2019 classification criteria for SLE [14]. In addition, ten age- and sex-matched healthy individuals who had no autoimmune disease, liver disease, or various systemic inflammatory diseases such as infection, diabetes mellitus, etc. were used as the controls in this study. Blood samples from all patients and controls were collected from the median cubital vein using an aseptic technique. The serum from individual samples was separated by centrifugation at 1,800*g* for 5 min, and then the level of ANA was confirmed using an immunofluorescence test kit (Euroimmun AG, Lübeck, Germany) according to the manufacturer’s instructions. Furthermore, serum samples were lyophilized until completely dry using a lyophilizer (Labcon, CA, USA) for performing further Western blot analysis.

### 2.2. Proteomics data retrieval

The data involved with serum/plasma proteins for the disease diagnosis and prognosis of SLE patients with the search terms including ‘serum protein in SLE’ or ‘plasma protein in SLE’ were retrieved from all published articles cited in PubMed and Science Direct database (2014–2020). All eligible serum/plasma proteins collected by the previous proteomics reports were defined as the expression alteration in only the SLE patients, not in healthy controls and other diseases involved with the change of protein level in serum/plasma, including liver disease, and systemic inflammatory disease.

### 2.3. Protein–protein interaction analysis

After the selection of proteomic data from previously published articles according to the aforementioned criteria, the altered serum/plasma proteins were used to predict the association network with the molecules related to the severity of SLE. This included C-reactive protein (CRP), complement C1q, C3, and C4 by Search Tool for the Retrieval of Interacting Genes/Proteins (STRING); version 11.0 (https://string-db.org/) with medium stringency/confidence level (score ≥0.400). This global network analysis was carried out relative to gene ontology (GO) terms based on the physical protein interactions from literature combined with the database of curated biological pathway knowledge.

### 2.4. Western blot analysis

The concentration of proteins derived from individual serum samples was initially measured by Bradford’s method using a Coomassie Plus (Bradford) assay kit (Thermo Scientific Pierce, IL, USA). An equal amount of protein in each sample (30 μg) was resolved in 12% SDS-PAGE and then transferred onto a nitrocellulose membrane (Whatman, Dassel, Germany) using a semidry transfer apparatus (Bio-Rad Laboratories, CA, USA) at 85 mA for 1 h. The membrane was stained with Ponceau S dye (Sigma–Aldrich, MO, USA) to verify equal protein loading and to serve as the loading control for subsequent quantitative intensity analysis. Subsequently, the membrane was destained with phosphate buffer saline (PBS), and non-specific bindings were blocked with 5% skim milk in PBS at 25 °C for 1 h. The membrane was then incubated with rabbit monoclonal anti-alpha-1 antitrypsin (Abcam, Cambridge, UK) diluted 1:2000 in 1% skim milk/PBS at 4 °C, overnight. After washing with PBS, the membrane was incubated with goat-anti-rabbit immunoglobulin G (IgG) H&L conjugated with horseradish peroxidase (HRP) (Abcam, Cambridge, UK) diluted 1:10,000 in 1% skim milk/PBS at 25 °C for 1 h. After washing with PBS, the bands of immune reactive protein were visualized by Clarity western enhanced chemiluminescence (ECL) substrate (Bio-Rad Laboratories, CA, USA) and autoradiography. The data of band intensity in each sample was obtained using ImageJ (National Institutes of Health (NIH), MD, USA).

### 2.5. Ethical issues

All patients provided written informed consent prior to participation. The study was approved by the UPHEC, Thailand (approval no. 2/143/62) in concordance with international guidelines including the Declaration of Helsinki, the Belmont Report, and ICH Good Clinical Practice.

### 2.6. Statistical analysis

The quantitative data were presented as mean ± standard deviation (SD). Comparison between the two groups and the relationship between two parameters were examined by Mann–Whitney *U* test and Spearman correlation following linear regression, respectively. Diagnostic performance was analyzed by the area-under-the-curve (AUC) of the receiver operating characteristic (ROC), with a p*-*value ≤ of 0.05 considered statistically significant. Statistical analyses were performed using SPSS (Version 25; IBM, NY, USA) and GraphPad Prism (Version 8; GraphPad Software, CA, USA).

## 3. Results

### 3.1. Systematic collection of proteomic data in SLE serum/plasma

All of the serum/plasma proteins that were altered in SLE patients were retrieved from previous proteomics studies using a literature search with the term ‘*serum/plasma proteins in SLE*’ from the PubMed and Science Direct databases since 2014 to 2020. Previous data in SLE patients based on statistical fold-change compared to healthy controls exhibited elevated 19 serum/plasma proteins. These included haptoglobin (*HP*), syndecan-1 (*SDC-1*), insulin-like growth factor binding protein II (*IGFBP2*), tumor necrosis factor receptor type II (*TNFR2*), tyrosine-protein kinase receptor UFO (*Axl* ), fibrinogen gamma chain (*FGG*), protein S100-A9 (*S100A9*), marginal zone B and B1 cell-specific protein (*MZB1*), alpha-1-acid glycoprotein 1 (*ORM1*), alpha-1-acid glycoprotein 2 (*ORM2*), alpha-1-antitrypsin (*SERPINA1*), alpha-2-macroglobulin (*A2M*), apolipoprotein B (*APOB*), ceruloplasmin (*CP*), complement factor H (*CFH* ), hemoglobin subunit alpha-1 (*HBA-1*), hemoglobin subunit beta (*HBB*), retinol-binding protein (*RBP4*), and serotransferin (*TF* ). In contrast, 7 serum/plasma proteins were found to reduce including glutathione S-transferase kappa 1 isoform c (*GSTK1*), apolipoprotein A-1 (*APOA1*), histidinerich glycoprotein (*HRG*), hemopexin (*HPX*), albumin (*ALB*), clusterin (*CLU* ), and fibrinogen beta chain (*FGB*) (Table 1).

### 3.2. Selection of serum/plasma proteins for candidate biomarkers

**Fig. 1:**
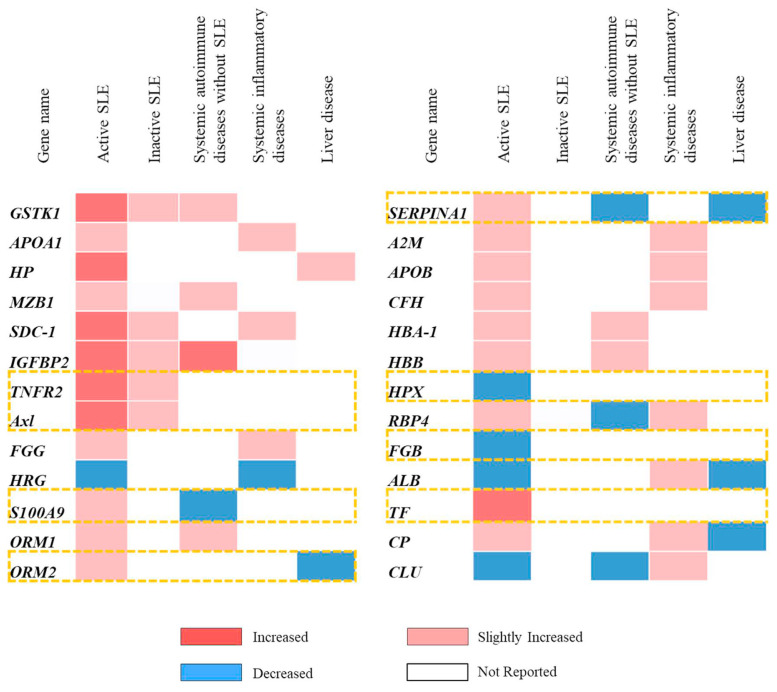
List of the candidate proteins in serum/plasma of SLE patients that were found the abnormal level in only SLE (active and/or inactive), not in other systemic autoimmune disease, systemic inflammatory disease, and liver disease (dot box) from 26 collected proteins in the published articles cited in PubMed and ScienceDirect database since 2014 to 2020.

The collected 26 serum/plasma proteins of SLE patients were subsequently selected to serve as the candidate biomarkers for specific prognosis of severity by following defined criteria, including only the encounter in SLE patients (active and/or inactive), not in other systemic autoimmune diseases such as rheumatoid arthritis, etc., or systemic inflammatory diseases such as diabetes mellitus, etc., and liver diseases. The proteins that had the altered level in serum/plasma of SLE patients with the contrast of other related diseases as aforementioned were also selected for further investigation. For example, alpha-1-antitrypsin (*SERPINA1*) was selected because there was an increased level in SLE patients, but was found to diminish in other systemic autoimmune diseases and liver diseases. The results demonstrated that tumor necrosis factor receptor type II (*TNFR2*), tyrosine-protein kinase receptor UFO (*Axl* ), protein S100-A9 (*S100A9*), alpha-1-acid glycoprotein 2 (*ORM2*), alpha-1-antitrypsin (*SERPINA1*), hemopexin (*HPX* ), fibrinogen beta chain (*FGB*), and serotransferrin (*TF* ) had properties based on the aforementioned criteria (Fig. 1). This suggests that these proteins may serve as specific candidate biomarkers for the prognosis of disease severity in SLE patients.

### 3.3. Association of the candidate serum proteins with molecules related to SLE severity

**Fig. 2:**
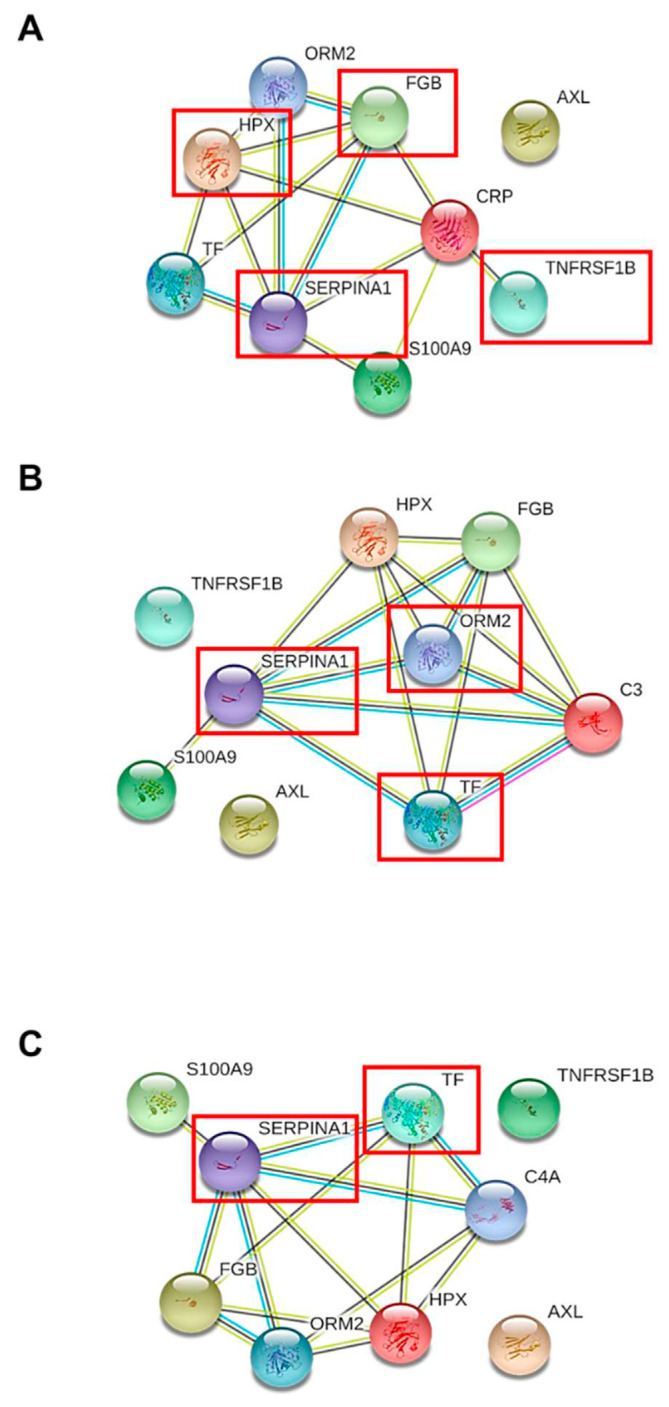
Bioinformatics analysis of protein–protein interaction between 8 candidate serum proteins in SLE patients with molecules related to disease severity consisting CRP (A), complement C3 (B), and C4 (C) by STRING tool, version 11.0 (http://string.embl.de/). Each interacting line color represented the interaction evidences contained curate database (blue), text mining (yellow), co-expression (black), and experimental determined (pink).

**Fig. 3:**
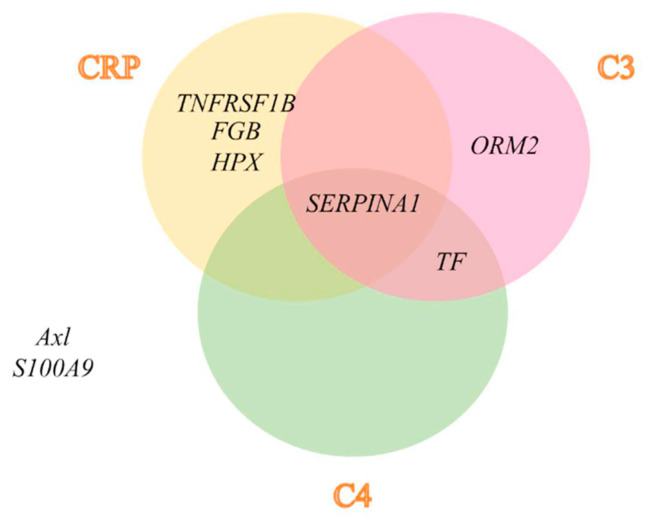
Diagram illustrating the association of 8 candidate proteins in serum/plasma of SLE patients with the severity molecules in this study including CRP, complement C3, and C4 (SERPINA1), both of C3 and C4 (TF), only CRP (TNFRSF1B, FGB, and HPX), only C3 (ORM2), and also had not associated with all of the severity molecules (Axl and S100A9), respectively after predicting analysis of the interaction by bioinformatics tool.

Tumor necrosis factor receptor type II (*TNFR2*), tyrosine-protein kinase receptor UFO (*Axl* ), protein S100-A9 (*S100A9*), alpha-1-acid glycoprotein 2 (*ORM2*), alpha-1-antitrypsin (*SERPINA1*), hemopexin (*HPX*), fibrinogen beta chain (*FGB*), and serotransferrin (*TF* ) were reported to vary in active SLE patients. However, there was no change of serum/plasma expression in other systemic autoimmune diseases, systemic inflammatory disease, or liver disease, which were then subjected to the protein network analysis STRING bioinformatics tool to investigate the association between these candidate biomarkers with CRP, complement C1q, C3, and C4. The integrative proteome network created by the STRING tool showed that tumor necrosis factor receptor type II (*TNFR2*), alpha-1-antitrypsin (*SERPINA1*), hemopexin (*HPX*), and fibrinogen beta chain (*FGB*) strongly interacted with CRP (Fig. 2A), alpha-1-acid glycoprotein 2 (*ORM2*), alpha-1-antitrypsin (*SERPINA1*), and serotransferrin (*TF* ), interacted strongly with complement C3 (Fig. 2B), while, alpha-1-antitrypsin (SERPINA1), and serotransferrin (TF) interacted strongly with complement C4 (Fig. 2C). From the results, alpha-1-antitrypsin (*SERPINA1*) was shown to relate with all inflammatory molecules in the current study, including CRP, complement C3, and C4 (Fig. 3), indicating that this serum/plasma protein had the closest association with the disease severity of SLE.

### 3.4. Validating the level of alpha-1-antitrypsin in the serum of SLE patients

**Fig. 4:**
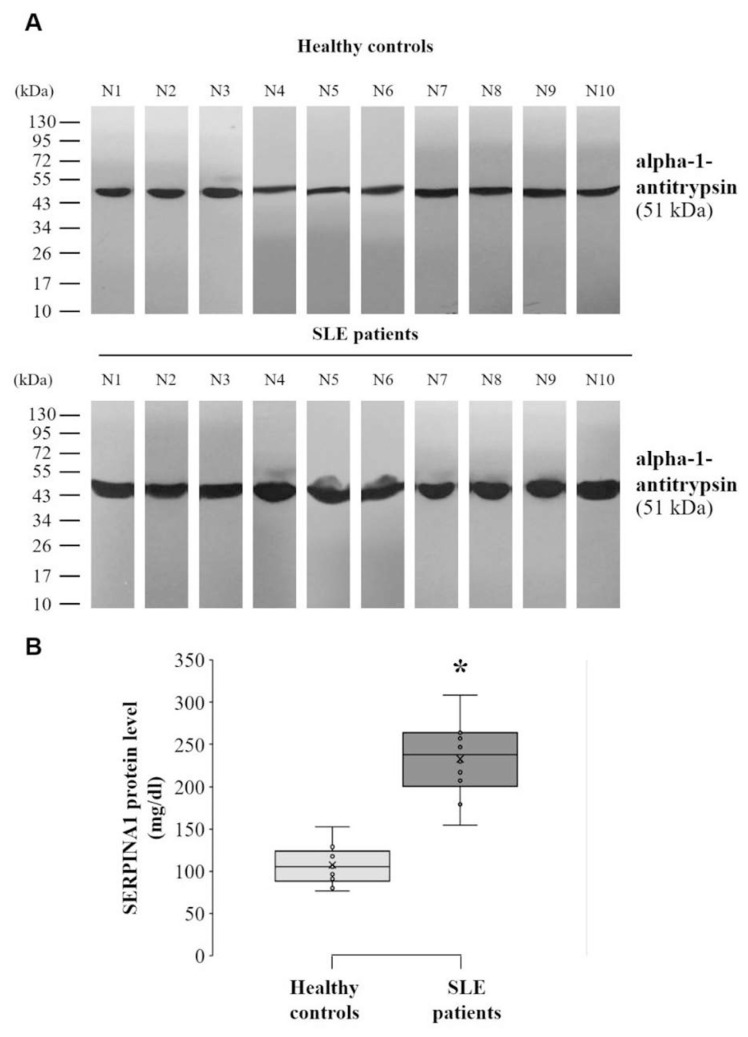
Level of serum alpha-1-antitrypsin (SERPINA1) in SLE patients (N = 10) was validated and compared to healthy controls (N = 10) by Western blot analysis. (A) An equal amount of protein extracted from individual sample (30 μg) were subjected to SDS-PAGE and followed by monoclonal antibody specific to alpha-1-antitrypsin (SERPINA1). (B) Band intensity of alpha-1-antitrypsin (SERPINA1) was measured using ImageJ software and normalized with total protein in each sample of SLE patients and healthy controls. The data in each bar was obtained from 10 independent participants and represented as mean ± SD. * p < 0.05 vs. controls.

The amount of total protein from individual serum samples in SLE patients and healthy controls was adjusted and then incubated with a monoclonal antibody specific to alpha-1-antitrypsin for validation of alpha-1-antitrypsin level in serum sample of the patients compared to controls. The serum level of alpha-1-antitrypsin in SLE patients (233.02 ± 14.32 mg/dl) was shown to be significantly higher than in healthy controls (107.54 ± 7.38 mg/dl) (p < 0.05) (Fig. 4).

### 3.5. Correlation between alpha-1-antitrypsin level and ANA titer in serum sample of SLE patients

**Fig. 5:**
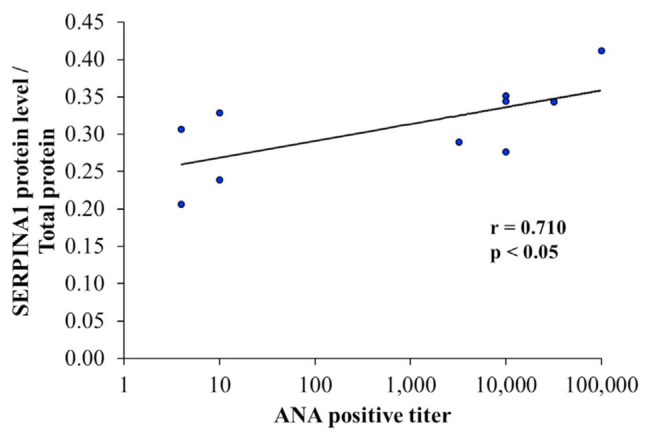
The correlation analysis of serum alpha-1-antitrypsin (SERPINA1) level with ANA positive titer from 10 independent participants of SLE patients.

ANA is currently indicated as a marker for laboratory diagnosis and also the prognosis of SLE severity. Therefore, ANA titer was applied to examine the correlation with the level of alpha-1-antitrypsin level in serum samples of SLE patients. The analysis of statistic correlation revealed that the serum level of alpha-1-antitrypsin was positively related to ANA positive titer (r = 0.710, p < 0.05) in SLE patients (Fig. 5).

### 3.6. Diagnostic power of serum alpha-1-antitrypsin level

**Fig. 6:**
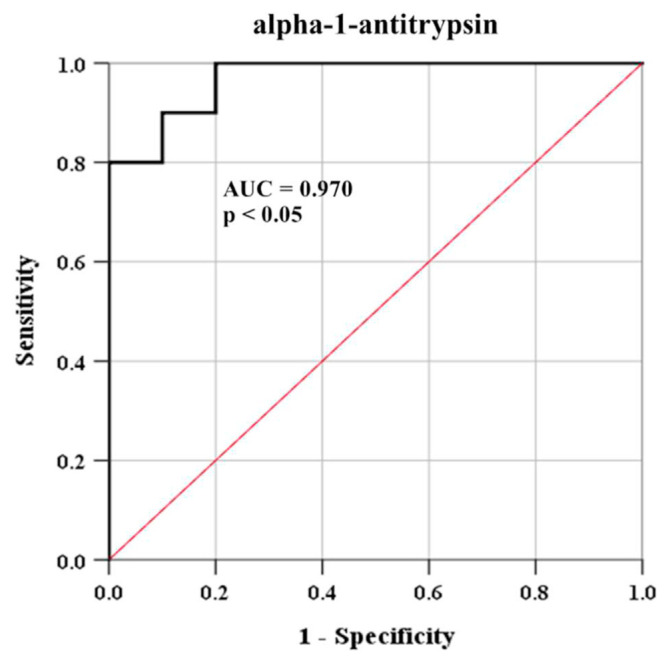
ROC curve in the evaluating diagnostic ability of serum alpha-1-antitrypsin (SERPINA1) in SLE patients (N = 10) and healthy controls (N = 10).

The diagnostic power of alpha-1-antitrypsin in serum samples was evaluated by ROC analysis. The area under the ROC curve after comparison between SLE patients and healthy controls was found at 0.970 (95% confidence interval; CI = 0.907–1.000, p < 0.05). For distinguishing serum alpha-1-antitrypsin level in SLE patients from healthy controls, the optimal cut-point of this protein was estimated by identification of value, which yielded the highest sensitivity and specificity. The cut-point of serum alpha-1-antitrypsin as 0.131/total serum protein was found to yield a sensitivity of 100% and specificity of 90% (Fig. 6).

## 4. Discussion

Literature searches of published articles concerning proteomics study from PubMed and Science-Direct between 2014 and 2020 [9–12,15–18] were applied to comprehensively collect various serum/plasma proteins in SLE patients. The data in these published articles indicated 26 proteins from SLE patients had an abnormal (increased or decreased) level compared to healthy controls (Table 1). In addition, the abnormal level of 8 from 26 collected serum/plasma proteins was suggested as a candidate serum/plasma biomarker for specific prognosis of SLE severity because these proteins had only been encountered in SLE patients, especially the active stage of the disease, or found a contrast level with other diseases involved with 1) the pathogenesis of SLE, including systemic autoimmune diseases and systemic inflammatory diseases, and 2) the abnormal production of circulatory proteins in liver diseases (Fig. 1). The candidate serum/plasma proteins, which were found in only SLE patients, consisted of tumor necrosis factor receptor type II (*TNFR2*), tyrosine-protein kinase receptor UFO (*Axl* ), hemopexin (*HPX* ), fibrinogen beta chain (*FGB*), and serotransferrin (*TF* ) (Fig. 1). Although the level of protein S100-A9 (*S100A9*), alpha-1-acid glycoprotein 2 (*ORM2*), alpha-1-antitrypsin (*SERPINA1*) was altered in various diseases other than SLE, these proteins were selected for the candidate biomarker because of the establishing contrast level in serum/plasma between SLE patients and other related diseases, i.e., the level of alpha-1-antitrypsin (*SERPINA1*) was found to increase in SLE, but diminish in other systemic autoimmune diseases and liver diseases.

Acute-phase protein CRP, complement C3 and C4 have been reported as crucial molecules related to disease severity in multi-organ damage of SLE patients via an inflammatory process and immune attack to the organ tissues, respectively [19,20]. Moreover, these molecules have been found in the circulatory system and could monitor disease severity progression in patients [6,7]. The molecules of CRP, complement C3 and C4, were applied to analyze the association with 8 candidate proteins in serum/plasma specific to SLE patients from the selection in the aforementioned criteria. Bioinformatics has recently been established as an important tool for visualizing, analyzing and retrieving the structure, kinetic and interaction of proteins from a large number of proteomic data sets. In addition, the identification of protein–protein interaction could provide understanding and insight into the cellular dynamics and functions involved in the pathogenesis of the disease [21]. Bioinformatics tool was applied to analyze the protein–protein interaction of the candidate biomarker with severity molecules in the circulatory system of SLE. STRING is a useful database and online resource (http://string-db.org) for the prediction of protein–protein association encoded by the known gene from many sources comprising experimental data, computational analyzing, and text collection. The STRING tool can generate an association among proteins based on raw data repositories as well as formalize pathways retrieved from protein databases and literature searches. The confidence of interaction score in the network was also supported to achieve a strong protein–protein interaction [22,23]. The protein network analysis revealed that alpha-1-antitrypsin strongly interacted with CRP, complement C3 and C4 with curate database, text mining, and/or co-expression (Figs. 2 and 3). Nevertheless, the remaining candidate serum proteins consisting of serotransferrin, tumor necrosis factor receptor type II, fibrinogen beta chain, hemopexin, alpha-1-acid glycoprotein 2, tyrosine-protein kinase receptor UFO, and protein S100-A9 were found to not be associated with all of the circulatory molecules related to SLE severity in this study (CRP, complement C3 and C4) (Fig. 3). Therefore, the bioinformatics data implied alpha-1-antitrypsin was the most altered serum biomarker for the prognosis of disease severity in SLE patients.

Alpha-1-antitrypsin is a human alpha globulin glycoprotein encoded by the *SERPINA1* gene and belonging to the serpin superfamily (inhibitor of serine protease). It is mostly produced by hepatocytes and by other cells to a lesser extent, such as macrophages/monocytes, neutrophil, pancreas, enterocytes, and some cancer cells [24,25]. In addition, alpha-1-antitrypsin can be secreted into the bloodstream with a half-life of 4–5 days. It has been found to be the most abundant serine protease inhibitor in the human circulatory system [26,27]. Because alpha-1-antitrypsin can inhibit serine protease, neutrophil elastase, and other enzymes in inflammatory cells, the biological roles of this protein are intimately involved with the inflammatory process. Moreover, alpha-1-antitrypsin could regulate the pathways of fibrinolysis, complement and coagulation [23,24], and its concentration in the bloodstream was also rapidly raised during inflammation to respond to the process [24,28], suggesting an acute-phase protein. Based on supporting data from previous and present studies, alpha-1-antitrypsin in SLE patients was then validated the serum level by Western blot analysis. The results demonstrated that serum alpha-1-antitrypsin was increased in SLE patients as compared to healthy controls (Fig. 4). Our findings were consistent with previous data that showed an increased plasma concentration of alpha-1-antitrypsin in SLE patients with severe cardiovascular manifestation than without- and healthy controls [29]. Moreover, various forms of circulating alpha-1-antitrypsin have exhibited the biological role involved with the inflammatory process, immune response and also found in patients. For example, the cleaved form was able to activate neutrophil for immunity enhancement, while S-nitrosylated form could induce pro-inflammatory cytokines and inducible nitric oxide synthase (iNOS) secreted from activated macrophages, oxidized and alpha-1-antitrypsin/immunoglobulin complex form was shown in patients with inflammatory diseases and systemic autoimmune disease, respectively [30,31]. Moreover, the production of alpha-1-antitrypsin in various cells such as hepatocytes could be upregulated by cytokines, especially interleukin-6 (IL-6) secreted from inflammatory cells [32]. IL-6 from monocytes was found to significantly alter the glycosylation for the production of the glycosylated form of alpha-1-antitrypsin related to several inflammatory conditions and diseases [33]. Additionally, the severity of SLE in the active stage was also demonstrated to be associated with the dysregulation of IL-6 inflammatory cytokine secretion in patients [34]. Hence, the level of alpha-1-antitrypsin in the serum sample had profound relevance to the pathogenic mechanism of disease severity in SLE patients, especially the inflammatory process and possibly through IL-6 induction, which should be investigated in further study.

The positive titer of ANA over a laboratory reference range is established as one of the important criteria for diagnosis and classification of SLE according to the 2019 EULAR/ACR criteria [14]. Additionally, immunofluorescence ANA testing has been indicated as the gold standard of laboratory investigation for SLE diagnosis, severity prognosis, and therapeutic monitoring [35]. The level of serum alpha-1-antitrypsin in SLE patients was applied to analyze the statistic correlation with the aforementioned gold standard method. The results exhibited the positive correlation between alpha-1-antitrypsin level to the ANA positive titer with high linearity (r = 0.710) (Fig. 5). Moreover, the level of alpha-1-antitrypsin with cut-point as 0.131/total serum protein was found to have a high sensitivity (100%) and specificity (90%) in the discrimination of SLE patients from healthy controls by ROC curve analysis (Fig. 6). It is possible that alpha-1-antitrypsin could serve as a novel serum biomarker for a rapid, specific, and reliable prognostic tool of disease severity associated with organ damage in SLE patients via the process of complementary and inflammatory activations. Previous study also demonstrated that alpha-1-antitrypsin was markedly present in the urine samples of SLE patients with active renal conditions for serving as a potential biomarker of lupus nephritis activity, which possibly had involvement with proteinuria in the patients [36]. Furthermore, the therapeutic potential of alpha-1-antitrypsin was recently evaluated and revealed a promising result with a safety profile on various autoimmune diseases such as type 1 diabetes, rheumatoid arthritis, and systemic lupus erythematosus, which is possibly caused by an anti-inflammatory effect of the protein [37].

After the formation of an immune complex between autoantibodies with soluble nuclear antigen, this complex can deposit multi-organ tissues and also activate the classical pathway of the complementary system. The activation of this pathway ultimately results in the formation of a membrane attack complex (MAC) by the assembly of complement C5b, C6, C7, C8, and C9 for the lysis of target cells [20]. Additionally, the complement cascade can produce complement C5a, which is a potent chemotaxis factor for inducing the migration of inflammatory cells consisting of neutrophils, monocytes, and other granulocytes into damaged organ tissues. Furthermore, these inflammatory cells are subsequently stimulated at the site of inflammation for cell–cell adhesion, production and secretion of various inflammatory mediators such as acute-phase proteins, and pro-inflammatory cytokines, resulting in further systemic organ injury and eventual failure [20,38]. Recently, the disease severity in multi-organ tissues of SLE patients was monitored by the level of CRP as an acute-phase protein, complement, especially C3 and C4 in serum/plasma, and also the finding of immune complex accumulated in organs [6,7]. These current laboratory investigations revealed several defects including the low sensitivity of detection and specificity to the patients [39]. The present study had limitations for obtaining a larger spectrum of SLE patients with various levels of IL-6 cytokine and stages of severity consisting of the non-active stage, active stage with nephritis, arthritis, or photosensitivity, etc. because there have been confined cases of SLE patients in Thailand [2]. These clinical stages of SLE severity were previously found to be associated with the different phenotypes of alpha-1 antitrypsin [37], suggesting that the validation of alpha-1 antitrypsin level in SLE’ serum should further consider the clinical association and also the IL-6 level of the patients. From our results, an increased level of serum alpha-1-antitrypsin specific in SLE patients associated with the pathogenic mechanisms of severity might indicate the need for an alternative laboratory investigation concerning the significance of prognosis and monitoring of disease. Furthermore, the increased level of serum alpha-1 antitrypsin in SLE patients might provide beneficial information for the potential management of disease severity as well as both prevention and treatment, which is the translational medical development of SLE. This data can be also used as a guideline for functional studies to better understand the pathogenic mechanisms in the disease progression of SLE leading to multi-organ injury.

## 5. Conclusion

The present study demonstrated that alpha-1-antitrypsin is a serum/plasma protein that specifically increased levels in SLE patients and dramatically showed the association with all of the severity molecules containing CRP, complement C3, and C4 by a systematic review of proteome data-based approach, and integrative bioinformatics analysis, respectively. In addition, the analysis of Western blot and statistic correlation showed a higher level of this protein in SLE patients compared to healthy controls as well as a strong correlation with the ANA titer in another laboratory diagnosis. Furthermore, the cut-point of serum alpha-1-antitrypsin at 0.131/total serum protein could discriminate patients from controls with high sensitivity and specificity. Taken together, the level of alpha-1-antitrypsin has the potential to be applied as a novel serum biomarker for further supporting the prognosis and monitoring of the change in SLE severity specific to patients.

## Figures and Tables

**Table 1:** List of serum proteins in SLE patients retrieved from all published articles cited in PubMed and ScienceDirect database since 2014 to 2020.

No.	Protein name	Gene name	Reference	Level (Fold change to healthy control)	Source
1	Glutathione S-transferase kappa 1 isoform c	*GSTK1*	Wang L et al., 2012 [15]	Decreased (0.5)	Serum, PBMC
2	Apolipoprotein A-1	*APOA1*	Kazemipour N et al., 2015 [9]	Decreased (0.8)	Serum
3	Haptoglobin	*HP*		Increased (1.3)	Serum, Urine
4	Syndecan-1	*SDC-1*	Kim KJ et al., 2015 [16]	Increased (1.9)	Serum
5	Insulin-like growth factor binding protein II	*IGFBP2*	Mok CC et al., 2016 [17]	Increased (3.1)	Serum
6	Tumor necrosis factor receptor type II	*TNFR2*		Increased (3.9)	Serum, PBMC
7	Tyrosine-protein kinase receptor UFO	*Axl*		Increased (1.8)	Serum
8	Fibrinogen gamma chain	*FGG*	Zhong L et al., 2017 [10]	Increased (6.2)	Serum
9	Histidine-rich glycoprotein	*HRG*		Decreased (1.0)	Serum
10	Protein S100-A9	*S100A9*		Increased (1.0)	Serum, PBMC
11	Marginal zone B and B1 cell-specific protein	*MZB1*	Miyagawa-Hayashino A et al., 2018 [18]	Increased (2.5)	Serum, B-cell, Lymph node biopsy
12	Albumin	*ALB*	Madda R et al., 2018 [11] and 2019 [12]	Decreased (0.8^12^)	Serum
13	Alpha-1-acid glycoprotein 1	*ORM1*		Increased (2.9^11^)	Serum
14	Alpha-1-acid glycoprotein 2	*ORM2*		Increased (2.3^11^)	Serum
15	Alpha-1-antitrypsin	*SERPINA1*		Increased (3.0^12^ – 3.2^11^)	Serum, Urine, Renal biopsy
16	Alpha-2-macroglobulin	*A2M*		Increased (3.8^12^)	Serum, Urine, Renal biopsy
17	Apolipoprotein B	*APOB*		Increased (3.2^12^–4^11^)	Serum
18	Ceruloplasmin	*CP*		Increased (2.9^12^–3.6^11^)	Serum, Urine
19	Clusterin	*CLU*		Decreased (0.4^11^)	Serum, Urine, Renal biopsy
20	Complement factor H	*CFH*		Increased (3.6^12^)	Serum
21	Fibrinogen beta chain	*FGB*		Decreased (0.6^12^)	Serum
22	Hemoglobin subunit alpha-1	*HBA-1*		Increased (5.3^12^)	Serum
23	Hemoglobin subunit beta	*HBB*		Increased (2.6^11^–3.8^12^)	Serum
24	Hemopexin	*HPX*		Decreased (0.2^11^)	Serum
25	Retinol-binding protein	*RBP4*		Increased (4.1^12^)	Serum
26	Serotransferrin	*TF*		Increased (2.3^11^)	Serum

## Abbreviations

*A2M*: Alpha-2-macroglobulin
*ACR*: American College of Rheumatology
*ALB*: Albumin
ANA: Anti-nuclear antibody
*APOA1*: Apolipoprotein A-1
*APOB*: Apolipoprotein B
*Axl*: Tyrosine-protein kinase receptor UFO
AUC: Area-under-the-curve
*CFH*: Complement factor H
*CLU*: Clusterin
*CP*: Ceruloplasmin
CRP: C-reactive protein
ECL: Enhanced chemiluminescence
ECL: Enhanced chemiluminescence
EULAR: European League Against Rheumatism
*FG*: Fibrinogen gamma chain
*FGB*: Fibrinogen beta chain
*GO*: Gene ontology
*GSTK1*: S-transferase kappa 1 isoform c
*HBA-1*: Hemoglobin subunit alpha-1
*HBB*: Hemoglobin subunit beta
*HP*: Haptoglobin
*HPX*: Hemopexin
*HRG*: Histidine-rich glycoprotein
HRP: Horseradish peroxidase
*IGFBP2*: Insulin-like growth factor binding protein II
IL-6: Interleukin-6
iNOS: Inducible nitric oxide synthase
MAC: Membrane attack complex
*MZB1*: Marginal zone B and B1 cell-specific protein
NIH: National Institutes of Health
*ORM1*: Alpha-1-acid glycoprotein 1
*ORM2*: Alpha-1-acid glycoprotein 2
PBMC: Peripheral blood mononuclear cell
PBS: Phosphate buffer saline
RBP4: Retinol-binding protein
ROC: Receiver operating characteristic
SD: Standard deviation
UPHEC: University of Phayao Human Ethics Committee, Thailand.
*S100A9*: Protein S100-A9
*SDC-1*: Syndecan-1
*SERPINA1*: Alpha-1-antitrypsin
SLE: Systemic lupus erythematosus
STRING: Search Tool for the Retrieval of Interacting Genes/Proteins
*TF*: Serotransferin
*TNFR2*: Tumor necrosis factor receptor type II

## References

[1] Stojan G Petri M Epidemiology of systemic lupus erythematosus: an update Curr Opin Rheumatol 2018 30 2 144 50 10.1097/BOR.0000000000000480 29251660 PMC6026543

[2] Ratanasiripong NT Ratanasiripong P Predictive factors of quality of life among systemic lupus erythematosus patients in Thailand: a web-based cross-sectional study Qual Life Res 2020 29 9 2415 23 10.1007/s11136-020-02494-6 32270369

[3] Robson MG Walport MJ Pathogenesis of systemic lupus erythematosus (SLE) Clin Exp Allergy 2001 31 5 678 85 10.1046/j.1365-2222.2001.01147.x 11422126

[4] Putterman C Caricchio R Davidson A Perlman H Systemic lupus erythematosus Clin Dev Immunol 2012 2012 437282 10.1155/2012/437282 23049599 PMC3459357

[5] Gurevitz SL Snyder JA Wessel EK Frey J Williamson BA Systemic lupus erythematosus: a review of the disease and treatment options Consult Pharm 2013 28 2 110 21 10.4140/TCP.n.2013.110 23395811

[6] Kuhn A Bonsmann G Anders HJ Herzer P Tenbrock K Schneider M The diagnosis and treatment of systemic lupus erythematosus Dtsch Arztebl Int 2015 112 25 423 32 10.3238/arztebl.2015.0423 26179016 PMC4558874

[7] Moroni G Depetri F Ponticelli C Lupus nephritis: when and how often to biopsy and what does it mean? J Autoimmun 2016 74 27 40 10.1016/j.jaut.2016.06.006 27349351

[8] Roşca A Modern aspects of the laboratory diagnosis of the systemic lupus erythematosus 2020

[9] Kazemipour N Qazizadeh H Sepehrimanesh M Salimi S Biomarkers identified from serum proteomic analysis for the differential diagnosis of systemic lupus erythematosus Lupus 2015 24 6 582 7 10.1177/0961203314558860 25391542

[10] Zhong L Liu J Zhou J Sun L Li C Li X Serum proteomics study reveals candidate biomarkers for systemic lupus erythematosus Int J Clin Exp Pathol 2017 10 10 10681 94 31966412 PMC6965801

[11] Madda R Lin SC Sun WH Huang SL Plasma proteomic analysis of systemic lupus erythematosus patients using liquid chromatography/tandem mass spectrometry with label-free quantification PeerJ 2018 6 e4730 10.7717/peerj.4730 29761050 PMC5947061

[12] Madda R Lin SC Sun WH Huang SL Differential expressions of plasma proteins in systemic lupus erythematosus patients identified by proteomic analysis J Microbiol Immunol Infect 2019 52 5 816 26 10.1016/j.jmii.2018.02.004 30170966

[13] Capecchi R Puxeddu I Pratesi F Migliorini P New biomarkers in SLE: from bench to bedside Rheumatology 2020 59 Supplement_5 v12 8 32911542 10.1093/rheumatology/keaa484 PMC7719038

[14] Aringer M Costenbader K Daikh D Brinks R Mosca M Ramsey-Goldman R 2019 European League against rheumatism/American College of Rheumatology classification criteria for systemic lupus erythematosus Arthritis Rheumatol 2019 71 9 1400 12 10.1002/art.40930 31385462 PMC6827566

[15] Wang L Dai Y Qi S Sun B Wen J Zhang L Comparative proteome analysis of peripheral blood mononuclear cells in systemic lupus erythematosus with iTRAQ quantitative proteomics Rheumatol Int 2012 32 3 585 93 10.1007/s00296-010-1625-9 21120503

[16] Kim KJ Kim JY Baek IW Kim WU Cho CS Elevated serum levels of syndecan-1 are associated with renal involvement in patients with systemic lupus erythematosus J Rheumatol 2015 42 2 202 9 10.3899/jrheum.140568 25512478

[17] Mok CC Ding HH Kharboutli M Mohan C Axl, ferritin, insulin-like growth factor binding protein 2, and tumor necrosis factor receptor type II as biomarkers in systemic lupus erythematosus Arthritis Care Res 2016 68 9 1303 9 10.1002/acr.22835 PMC5441892 26749069

[18] Miyagawa-Hayashino A Yoshifuji H Kitagori K Ito S Oku T Hirayama Y Increase of MZB1 in B cells in systemic lupus erythematosus: proteomic analysis of biopsied lymph nodes Arthritis Res Ther 2018 20 1 13 10.1186/s13075-018-1511-5 29382365 PMC5791339

[19] Szalai AJ C-reactive protein (CRP) and autoimmune disease: facts and conjectures Clin Dev Immunol 2004 11 3–4 221 6 10.1080/17402520400001751 15559367 PMC2486333

[20] Ballanti E Perricone C Greco E Ballanti M Di Muzio G Chimenti MS Complement and autoimmunity Immunol Res 2013 56 2–3 477 91 10.1007/s12026-013-8422-y 23615835

[21] Kumar C Mann M Bioinformatics analysis of mass spectrometry-based proteomics data sets FEBS Lett 2009 583 11 1703 12 10.1016/j.febslet.2009.03.035 19306877

[22] Mering Cv Huynen M Jaeggi D Schmidt S Bork P Snel B STRING: a database of predicted functional associations between proteins Nucleic Acids Res 2003 31 1 258 61 12519996 10.1093/nar/gkg034 PMC165481

[23] Szklarczyk D Gable AL Lyon D Junge A Wyder S Huerta-Cepas J STRING v11: protein–protein association networks with increased coverage, supporting functional discovery in genome-wide experimental datasets Nucleic Acids Res 2019 47 D1 D607 13 30476243 10.1093/nar/gky1131 PMC6323986

[24] Janciauskiene SM Bals R Koczulla R Vogelmeier C Kohnlein T Welte T The discovery of alpha1-antitrypsin and its role in health and disease Respir Med 2011 105 8 1129 39 10.1016/j.rmed.2011.02.002 21367592

[25] Heit C Jackson BC McAndrews M Wright MW Thompson DC Silverman GA Update of the human and mouse SERPIN gene superfamily Hum Genom 2013 7 22 10.1186/1479-7364-7-22 PMC3880077 24172014

[26] Dunlea DM Fee LT McEnery T McElvaney NG Reeves EP The impact of alpha-1 antitrypsin augmentation therapy on neutrophil-driven respiratory disease in deficient individuals J Inflamm Res 2018 11 123 34 10.2147/JIR.S156405 29618937 PMC5875399

[27] Hunt JM Tuder R Alpha 1 anti-trypsin: one protein, many functions Curr Mol Med 2012 12 7 827 35 10.2174/156652412801318755 22697349

[28] Stockley RA The multiple facets of alpha-1-antitrypsin Ann Transl Med 2015 3 10 130 10.3978/j.issn.2305-5839.2015.04.25 26207223 PMC4486914

[29] Svenungsson E Jensen-Urstad K Heimburger M Silveira A Hamsten A de Faire U Risk factors for cardiovascular disease in systemic lupus erythematosus Circulation 2001 104 16 1887 93 10.1161/hc4101.097518 11602489

[30] Lechowicz U Rudzinski S Jezela-Stanek A Janciauskiene S Chorostowska-Wynimko J Post-translational modifications of circulating alpha-1-antitrypsin protein Int J Mol Sci 2020 21 23 10.3390/ijms21239187 PMC7731214 33276468

[31] Kaner Z Engelman R Schuster R Rider P Greenberg D Av-Gay Y S-nitrosylation of alpha1-antitrypsin triggers macrophages toward inflammatory phenotype and enhances intra-cellular bacteria elimination Front Immunol 2019 10 590 10.3389/fimmu.2019.00590 31001247 PMC6454134

[32] Knoell DL Ralston DR Coulter KR Wewers MD Alpha 1-antitrypsin and protease complexation is induced by lipopolysaccharide, interleukin-1 β, and tumor necrosis factor-α in monocytes Am J Respir Crit Care Med 1998 157 1 246 55 9445306 10.1164/ajrccm.157.1.9702033

[33] McCarthy C Saldova R Wormald MR Rudd PM McElvaney NG Reeves EP The role and importance of glycosylation of acute phase proteins with focus on alpha-1 antitrypsin in acute and chronic inflammatory conditions J Proteome Res 2014 13 7 3131 43 10.1021/pr500146y 24892502

[34] Ding J Su S You T Xia T Lin X Chen Z Serum interleukin-6 level is correlated with the disease activity of systemic lupus erythematosus: a meta-analysis Clinics 2020 75 10.6061/clinics/2020/e1801 PMC7536892 33084768

[35] Kumagai S Hayashi N Immunofluorescence–still the ‘gold standard’ in ANA testing? Scand J Clin Lab Invest Suppl 2001 235 77 83 10.1080/003655101753352086 11712696

[36] Aggarwal A Gupta R Negi VS Rajasekhar L Misra R Singh P Urinary haptoglobin, alpha-1 anti-chymotrypsin and retinol binding protein identified by proteomics as potential biomarkers for lupus nephritis Clin Exp Immunol 2017 188 2 254 62 10.1111/cei.12930 28120479 PMC5383437

[37] Song S Alpha-1 antitrypsin therapy for autoimmune disorders Chronic Obstr Pulm Dis 2018 5 4 289 301 10.15326/jcopdf.5.4.2018.0131 30723786 PMC6361478

[38] Bolon B Cellular and molecular mechanisms of autoimmune disease Toxicol Pathol 2012 40 2 216 29 10.1177/0192623311428481 22105648

[39] Egner W The use of laboratory tests in the diagnosis of SLE J Clin Pathol 2000 53 6 424 32 10.1136/jcp.53.6.424 10911799 PMC1731203

